# Crystal structures of three 3-chloro-3-methyl-2,6-di­aryl­piperidin-4-ones

**DOI:** 10.1107/S2056989016020661

**Published:** 2017-01-06

**Authors:** R. Arulraj, S. Sivakumar, Manpreet Kaur, A. Thiruvalluvar, Jerry P. Jasinski

**Affiliations:** a Research and Development Centre, Bharathiar University, Coimbatore 641 046, Tamilnadu, India; b Department of Chemistry, Thiruvalluvar Arts and Science College, Kurinjipadi 607 302, Tamilnadu, India; c Department of Chemistry, Keene State College, 229 Main Street, Keene, NH 03435-2001, USA; d Principal, Government College for Women (Autonomous), Kumbakonam 612 001, Tamilnadu, India

**Keywords:** crystal structures, 3-chloro-3-methyl-2,6-diphenyl-piperidin-4-ones, hydrogen bonds, C—H⋯π inter­actions

## Abstract

The syntheses and crystal structure of 3-chloro-3-methyl-*r*-2,*c*-6-di­phenyl­piperidin-4-one, C_18_H_18_ClNO, (I), 3-chloro-3-methyl-*r*-2,*c*-6-di-*p*-tolyl­piperidin-4-one, C_20_H_22_ClNO, (II), and 3-chloro-3-methyl-*r*-2,*c*-6-bis­(4-chloro­phen­yl)piperidin-4-one, C_18_H_16_Cl_3_NO, (III), are described. In each structure, the piperidine ring adopts a chair conformation and dihedral angles between the mean planes of the phenyl rings are 58.4 (2), 73.5 (5) and 78.6 (2)° in (I), (II) and (III), respectively. In the crystals, mol­ecules are linked into *C*(6) chains by weak N—H⋯O hydrogen bonds and C—H⋯π inter­actions are also observed.

## Abstract 2

The syntheses and crystal structures of 3-chloro-3-methyl-*r*-2,*c*-6-di­phenyl­piperidin-4-one and two of its derivatives are described. In each structure, the piperidine ring adopts a chair conformation. In the crystals, mol­ecules are linked into *C*(6) chains by weak N—H⋯O hydrogen bonds and C—H⋯π inter­actions are also observed.

## Chemical context   

The piperidine ring is a ubiquitous structural feature of many alkaloid natural products and drug candidates: Watson *et al.* (2000 ▸) asserted that during a recent 10-year period there were thousands of piperidine compounds mentioned in clinical and preclinical studies. Piperidin-4-ones are reported to possess analgesic, anti-inflammatory, central nervous system (CNS), local anaesthetic, anti­cancer and anti­microbial activities (Perumal *et al.*, 2001 ▸; Dimmock *et al.*, 2001 ▸). As part of our ongoing structural studies of piperidin-4-ones (Arulraj *et al.*, 2016 ▸), the syntheses and crystal structures of three 3-chloro-3-methyl-2,6-di­aryl­piperidin-4-ones are now reported.
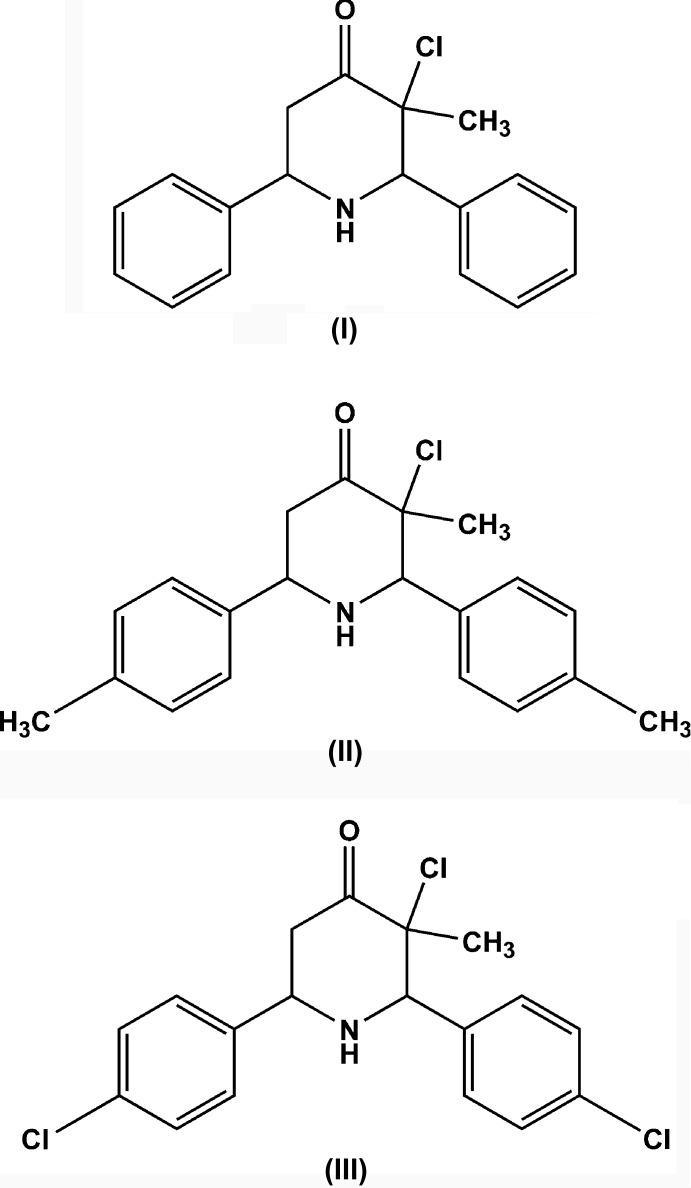


## Structural commentary   

**Figure 1:**
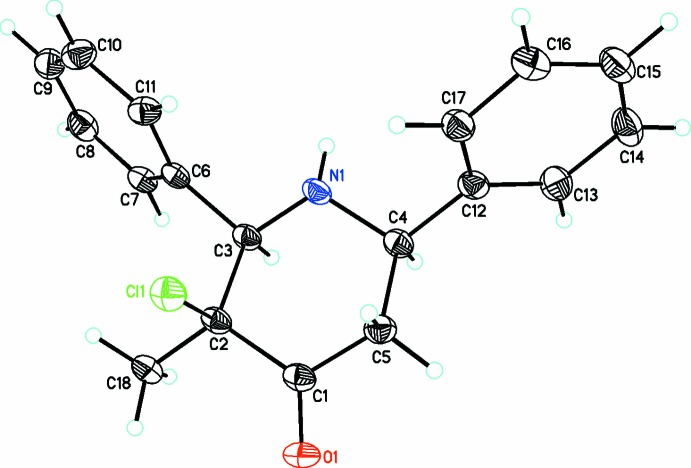
A view of the mol­ecular structure of (I), showing displacement ellipsoids drawn at the 30% probability level.

**Figure 2:**
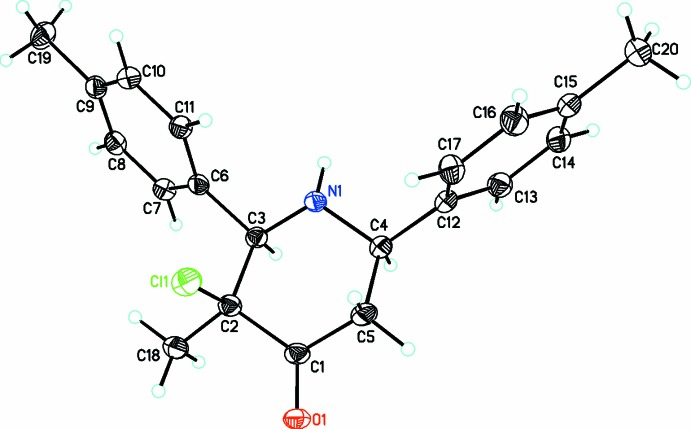
A view of the mol­ecular structure of (II), showing displacement ellipsoids drawn at the 30% probability level.

**Figure 3:**
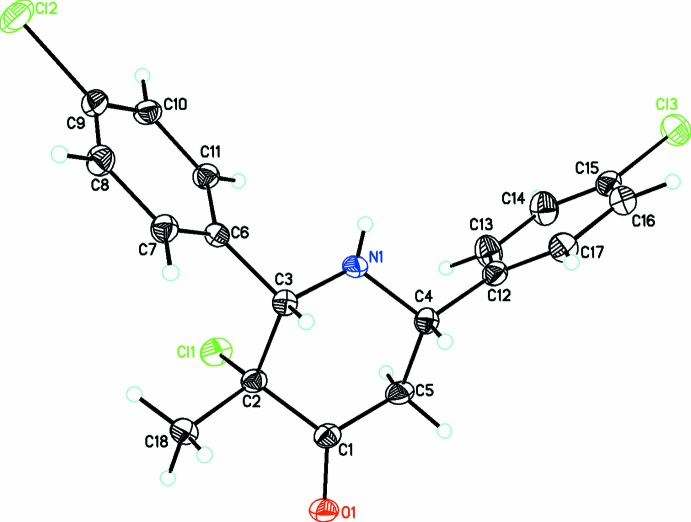
A view of the mol­ecular structure of (III), showing displacement ellipsoids drawn at the 30% probability level.

The title compound containing the 2,6-diaryl-piperidin-4-one moiety, C_18_H_18_NOCl, (I), crystallizes in the triclinic space group *P* 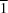 (Fig. 1 ▸) whereas compounds C_20_H_22_NOCl, (II) (Fig. 2 ▸) and C_18_H_16_NOCl_3_, (III) (Fig. 3 ▸) both crystallize in the ortho­rhom­bic space group *Pna*2_1_. The piperidin-4-one ring in all three compounds exhibits a distorted chair conformation [puckering parameters *Q* = 0.559 (3) Å (I), 0.568 (2) Å (II), 0.557 (3) Å (III); θ = 173.3 (3)° (I), 168.5 (2)° (II), 167.8 (3)° (III) and φ = 180 (2)° (I), 156.9 (12)° (II), 206.8 (13)° (III)]. The methyl substituent on position 3 of the piperidine ring takes up a syn-periplanar orientation [C18—C2—C1—O1 = −3.4 (3)° (I), −7.4 (3)° (II), 8.6 (4)° (III)] while the chloro substituent takes up an anti-clinical orientation [Cl1—C2—C1—O1 = 113.3 (2)° (I), 109.0 (2)° (II), −106.9 (3)° (III)] owing to the repulsion from a nearby oxygen atom. The phenyl rings bonded to the piperidine moiety occupy equatorial positions in all three compounds: the dihedral angles between the mean planes of the phenyl rings are 58.4 (2), 73.5 (5) and 78.6 (2)° in (I), (II) and (III), respectively. The increase in the dihedral angles between the phenyl rings from (I) to (III) might be attributed to the steric repulsion resulting from the substituents on the phenyl rings. The sum of bond angles around N1 in each structure [333.1° (I), 332.0° (II), 337.3° (III)] is consistent with *sp* ^3^ hybridization (Beddoes *et al.*, 1986 ▸).

## Supra­molecular features   

**Figure 4:**
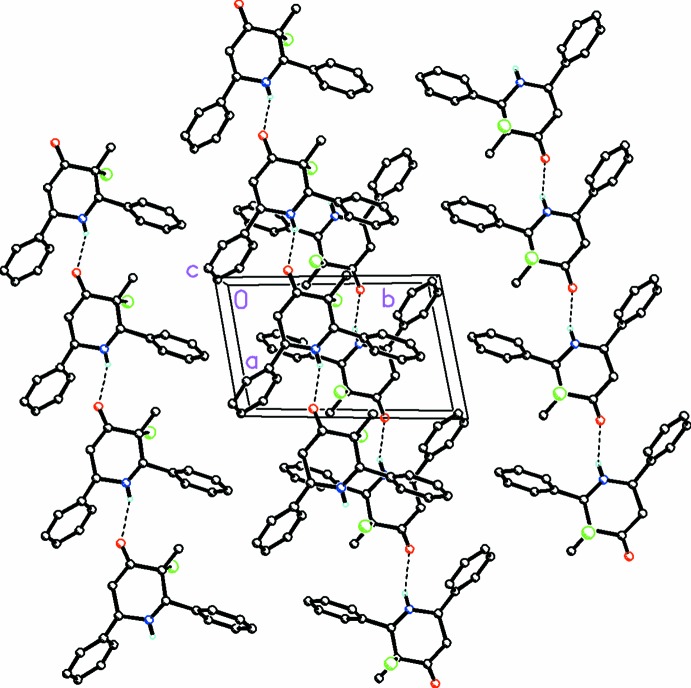
A partial view along the *c* axis of the crystal packing for (I), showing the chains formed along [100] by a weak N—H⋯O hydrogen bond. H atoms not involved in this weak hydrogen-bonding activity have been omitted for clarity.

For each structure, the crystal packing is influenced by weak N1—H1⋯O1 hydrogen bonds, forming infinite chains along the *a* axis direction (Figs. 4 ▸, 5 ▸ and 6 ▸). In (III), additional weak C10—H10⋯O1 inter­actions are observed. Weak C—H⋯π inter­actions are observed in all three compounds (Tables 1 ▸, 2 ▸ and 3 ▸). In all three compounds, π–π inter­actions must be extremely weak, with centroid–centroid separations greater than 4 Å.

## Database survey   

A search in the Cambridge Structural Database (CSD, Version 5.37, update February 2016; Groom *et al.*, 2016 ▸) for the 2,6-di­phenyl­piperidin-4-one skeleton gave 221 hits. Three closely related structures, *viz. c*-3,*t*-3-dimethyl-*r*-2,*c*-6-diphenyl-piperidin-4-one (CSD refcode: PUGNEL; Thenmozhi *et al.*, 2009 ▸); *r*-2,*c*-6-bis-(4-chloro­phen­yl)-3,3-di­methyl­piperidin-4-one (CSD refcode: OGEJEQ; Ilango *et al.*, 2008 ▸) and 3,3-dimethyl-cis-2,6-di-*p*-tolyl­piperidin-4-one (CSD refcode: PUFHAA; Gayathri *et al.*, 2009 ▸) may be briefly compared to the three structures reported here: the distorted chair conformations of the piperidine rings are also observed in PUGNEL, OGEJEQ and PUFHAA. The packing in (I),(II) and (III) and and PUGNEL, PUFHAA and OGEJEQ all feature N—H⋯O hydrogen bonds and C—H⋯π inter­actions. Both (III) and OGEJEQ also exhibit additional weak C—H⋯O inter­actions.

## Synthesis and crystallization   

A mixture of ammonium acetate (0.1 mol, 7.71 g), the respective aldehyde (0.2 mol) (benzaldehyde/*p*-methyl­benzaldehyde/*p*-chloro­benzaldehyde, 20.4 ml, 24.0 g and 28.1 ml) and 3-chloro-2-butanone (0.1 mol, 10.1 ml) in distilled ethanol was heated first to boiling. After cooling, the viscous liquid obtained was dissolved in diethyl ether (200 ml) and shaken with 100 ml concentrated hydro­chloric acid. The precipitated hydro­chloride of the 3-chloro, 3-methyl-*r*(6),*c*(6)-di­aryl­piperidin-4-one was removed by filtration and washed first with a 40 ml mixture of ethanol and diethyl ether (1:1) and then with diethyl ether to remove most of the coloured impurities. The base was liberated from an alcoholic solution by adding aqueous ammonia and then diluted with water. Each compound was recrystallized twice from distilled ethanol solution: single crystals of (I), (II) and (III) were obtained after two days.

**3-Chloro-3-methyl-** ***r*** **(2)**,***c*** **(6)-di­phenyl­piperidin-4-one, (C_18_H_18_ClNO) (I)** IR (KBr): 3333.64 (υN—H), 3063.43, 3007.40 (υC—H), 1713.51 (υC=O), 1602.76, 1495.15 (υC=C), 749.57 (υC—Cl) cm^−1. 1^H NMR (500 MHz, CDCl_3_): δ 7.41–7.16 (*m*, aromatic protons), 4.00–3.97 [*dd*, H(6) proton], 3.87 [*s*, H(2) proton] , 3.44–3.39 [*t*, H(5e) proton], 2.50–2.45 [*dd*, H(5a) proton], 1.66 (*s*, NH proton), 1.38 (*s*, CH_3_ proton). ^13^C NMR (CDCl_3_, 500 MHz): δ 202.69 (C=O), 142.27, 137.32 (aromatic *ipso* carbon atoms), 129.52–126.89 (aromatic carbon atoms), 72.02 (C-3 carbon), 69.88 (C-2 carbon), 61.49 (C-6 carbon), 45.60 (C-5 carbon), 22.25 (methyl carbon).

**3-Chloro-3-methyl-** ***r*** **(2)**,***c*** **(6)-di-p-tolyl-piperidin-4-one, (C_20_H_22_ClNO) (II)** IR (KBr): 3332.57 (υN—H), 3095.35, 3007.79 (υC—H), 1715.40 (υC=O), 1615.57, 1513.79 (υC=C), 738.68 (υC—Cl) cm^−1. 1^H NMR (500 MHz, CDCl_3_): δ 7.50–7.33 (*m*, aromatic protons), 4.06–4.03 [*dd*, H(6) proton], 3.93 [*s*, H(2) proton], 3.45–3.40 [*dd*, H(5e) proton], 2.54–2.51 [*dd*, H(5a) proton], 1.70 (*s*, NH proton), 1.43 (*s*, CH_3_ proton at C-3), 2.45 (*s*, CH_3_ protons attached to the phenyl ring). ^13^C NMR (CDCl_3_, 500 MHz): δ 203.07 (C=O), 139.32, 138.56, 138.01, 134.32 (aromatic *ipso* carbon atoms), 129.69–126.76 (aromatic carbon atoms), 72.16 (C-3 carbon), 69.62 (C-2 carbon), 61.18 (C-6 carbon), 45.58 (C-5 carbon), 21.37 (methyl carbon at C-3), 22.22 (methyl carbon atoms attached to the phenyl ring).

**3-Chloro-3-methyl-** ***r*** **(2)**,***c*** **(6)-bis­(p-chloro­phen­yl)piperidin-4-one, (C_18_H_16_Cl_3_NO) (III)** IR (KBr): 3325.87 (υN—H), 3047.68, 3009.09 (υC—H), 1715.63 (υC=O), 1596.88, 1491.72 (υC=C), 799.88 (υC—Cl) cm^−1. 1^H NMR (500 MHz, CDCl_3_): δ 7.50–7.33 (*m*, aromatic protons), 4.06–4.03 [*dd*, H(6) proton], 3.93 [*s*, H(2) proton], 3.45–.40 [*dd*, H(5e) proton], 2.54–2.51 [*dd*, H(5a) proton], 1.70 (*s*, NH proton), 1.43 (*s*, CH_3_ proton). ^13^C NMR (CDCl_3_, 500 MHz): δ 201.73 (C=O), 140.41, 135.41, 134.67, 133.93 (aromatic *ipso* carbon atoms), 130.55–128.04 (aromatic carbon atoms), 71.31 (C-3 carbon), 68.92 (C-2 carbon), 60.54 (C-6 carbon), 45.24 (C-5 carbon), 21.92 (methyl carbon).

## Refinement   

Crystal data, data collection and structure refinement details are summarized in Table 4 ▸. In (I), all H atoms were placed in their calculated positions and then refined using a riding model with bond lengths of 0.95 or 1.0 Å (CH), 0.99 Å (CH_2_), 0.98 Å (CH_3_) or 0.83 Å (NH). In (II) and (III), atom H1 was located in a difference map and refined isotropically. Isotropic displacement parameters for all these atoms in (I), (II) and (III) were set to 1.2 (CH, CH_2_) or 1.5 (CH_3_) times *U*_eq_ of the parent atom. Idealized methyl groups were refined as rotating groups. The refinement for (III) showed some parameter oscillation, and convergence was achieved with the use of a DAMP card.

## Supplementary Material

Crystal structure: contains datablock(s) I, II, III. DOI: 10.1107/S2056989016020661/hb7647sup1.cif

Structure factors: contains datablock(s) I. DOI: 10.1107/S2056989016020661/hb7647Isup2.hkl

Structure factors: contains datablock(s) II. DOI: 10.1107/S2056989016020661/hb7647IIsup3.hkl

Structure factors: contains datablock(s) III. DOI: 10.1107/S2056989016020661/hb7647IIIsup4.hkl

CCDC references: 1524979, 1524978, 1524977

Additional supporting information:  crystallographic information; 3D view; checkCIF report

## Figures and Tables

**Figure 5:**
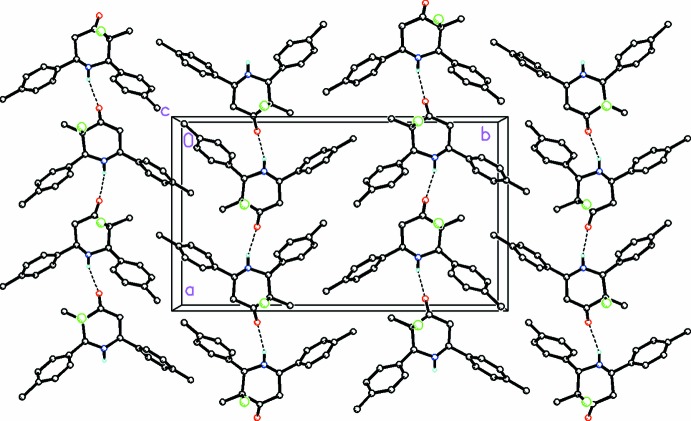
A partial view along the *c* axis of the crystal packing for (II) showing the chains formed along [100] by a weak N—H⋯O hydrogen bond. H atoms not involved in this weak hydrogen-bonding activity have been omitted for clarity.

**Figure 6:**
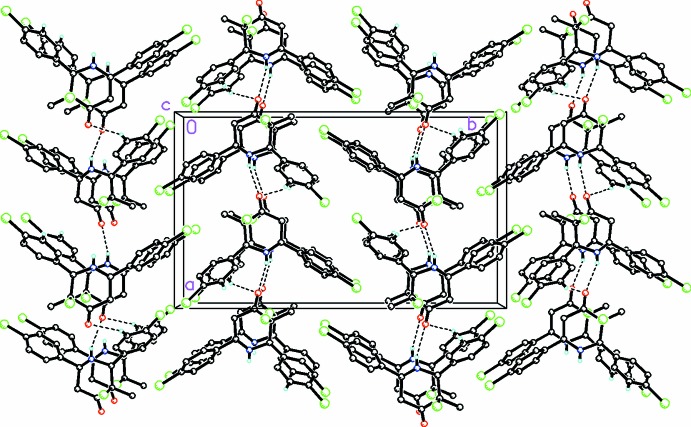
A partial view along the *c* axis of the crystal packing for (III) showing the chains formed along [100] by a single weak N—H⋯O inter­action, which is consolidated by a C—H⋯O bond. H atoms not involved in this weak hydrogen-bonding activity have been omitted for clarity.

**Table 1:** Hydrogen-bond geometry (Å, °) for (I) *Cg*2 and *Cg*3 are the centroids of the C6–C11 and C12–C17 rings, respectively.

*D*—H⋯*A*	*D*—H	H⋯*A*	*D*⋯*A*	*D*—H⋯*A*
N1—H1⋯O1^i^	0.83 (3)	2.49 (3)	3.257 (3)	154 (3)
C9—H9⋯*Cg*3^ii^	0.95	2.97	3.662 (3)	131
C15—H15⋯*Cg*2^iii^	0.96	2.98	3.861 (3)	155
C18—H18*A*⋯*Cg*2^iv^	0.98	2.73	3.497 (3)	136

Symmetry codes: (i) 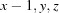; (ii) 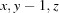; (iii) 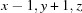; (iv) 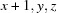.

**Table 2:** Hydrogen-bond geometry (Å, °) for (II) *Cg*2 and *Cg*3 are the centroids of the C6–C11 and C12–C17 rings, respectively.

*D*—H⋯*A*	*D*—H	H⋯*A*	*D*⋯*A*	*D*—H⋯*A*
N1—H1⋯O1^i^	0.85 (3)	2.27 (3)	3.057 (2)	154 (3)
C18—H18*A*⋯*Cg*3^ii^	0.98	2.92	3.686 (3)	135
C20—H20*A*⋯*Cg*2^iii^	0.97	2.81	3.724 (3)	156

Symmetry codes: (i) 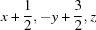; (ii) 
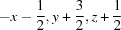
; (iii) 
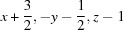
.

**Table 3:** Hydrogen-bond geometry (Å, °) for (III) *Cg*3 is the centroid of the C12–C17 ring.

*D*—H⋯*A*	*D*—H	H⋯*A*	*D*⋯*A*	*D*—H⋯*A*
N1—H1⋯O1^i^	0.74 (3)	2.40 (3)	3.071 (3)	151 (3)
C10—H10⋯O1^ii^	0.95	2.56	3.374 (3)	144
C18—H18*C*⋯*Cg*3^iii^	0.98	2.98	3.725 (3)	134

Symmetry codes: (i) 
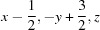
; (ii) 
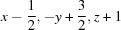
; (iii) 
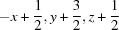
.

**Table 4:** Experimental details

	(I)	(II)	(III)
Crystal data			
Chemical formula	C_18_H_18_ClNO	C_20_H_22_ClNO	C_18_H_16_Cl_3_NO
*M*_r_	299.78	327.83	368.67
Crystal system, space group	Triclinic, *P* 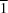	Orthorhombic, *P* *n* *a*2_1_	Orthorhombic, *P* *n* *a*2_1_
Temperature (K)	173	173	173
*a*, *b*, *c* (Å)	6.7150 (6), 10.9591 (13), 11.1704 (10)	13.0578 (2), 22.6513 (4), 5.93756 (8)	13.2430 (4), 22.3945 (6), 5.81947 (14)
α, β, γ (°)	72.162 (9), 79.721 (7), 76.873 (8)	90, 90, 90	90, 90, 90
*V* (Å^3^)	756.80 (14)	1756.19 (5)	1725.88 (8)
*Z*	2	4	4
Radiation type	Cu *K*α	Cu *K*α	Cu *K*α
μ (mm^−1^)	2.21	1.94	4.83
Crystal size (mm)	0.26 × 0.22 × 0.06	0.32 × 0.18 × 0.08	0.34 × 0.14 × 0.14

Data collection			
Diffractometer	Rigaku Oxford Diffraction	Agilent Xcalibur, Eos, Gemini	Agilent Xcalibur, Eos, Gemini
Absorption correction	Multi-scan *CrysAlis PRO* (Agilent, 2014 ▸)	Multi-scan *CrysAlis PRO* (Agilent, 2014 ▸)	Multi-scan *CrysAlis PRO* (Agilent, 2014 ▸)
*T*_min_, *T*_max_	0.609, 1.000	0.724, 1.000	0.646, 1.000
No. of measured, independent and observed [*I* > 2σ(*I*)] reflections	4920, 2847, 2456	11595, 2966, 2873	12474, 2602, 2494
*R*_int_	0.030	0.050	0.033
(sin θ/λ)_max_ (Å^−1^)	0.615	0.615	0.615

Refinement			
*R*[*F* ^2^ > 2σ(*F* ^2^)], *wR*(*F* ^2^), *S*	0.057, 0.168, 1.05	0.034, 0.087, 1.07	0.032, 0.084, 1.02
No. of reflections	2847	2966	2602
No. of parameters	195	214	212
No. of restraints	0	1	1
H-atom treatment	H atoms treated by a mixture of independent and constrained refinement	H atoms treated by a mixture of independent and constrained refinement	H atoms treated by a mixture of independent and constrained refinement
Δρ_max_, Δρ_min_ (e Å^−3^)	0.68, −0.29	0.24, −0.21	0.45, −0.23
Absolute structure	–	Flack *x* determined using 1017 quotients [(*I* ^+^)−(*I* ^−^)]/[(*I* ^+^)+(*I* ^−^)] (Parsons *et al.*, 2013 ▸)	Flack *x* determined using 695 quotients [(*I* ^+^)−(*I* ^−^)]/[(*I* ^+^)+(*I* ^−^)] (Parsons *et al.*, 2013 ▸)
Absolute structure parameter	–	−0.010 (13)	0.135 (13)

Computer programs: *CrysAlis PRO* (Agilent, 2014 ▸), *SHELXT* (Sheldrick, 2015*a* ▸), *SHELXL* (Sheldrick, 2015*b* ▸) and *OLEX2* (Dolomanov *et al.*, 2009 ▸).

## supplementary crystallographic information

### (I) 3-Chloro-3-methyl-r-2,c-6-diphenylpiperidin-4-one Crystal data

**Table:**

C_18_H_18_ClNO	*Z* = 2
*M**_r_* = 299.78	*F*(000) = 316
Triclinic, *P*1	*D*_x_ = 1.316 Mg m^−^^3^
*a* = 6.7150 (6) Å	Cu *K*α radiation, λ = 1.54184 Å
*b* = 10.9591 (13) Å	Cell parameters from 1924 reflections
*c* = 11.1704 (10) Å	θ = 4.2–71.4°
α = 72.162 (9)°	µ = 2.21 mm^−^^1^
β = 79.721 (7)°	*T* = 173 K
γ = 76.873 (8)°	Plate, colourless
*V* = 756.80 (14) Å^3^	0.26 × 0.22 × 0.06 mm

### (I) 3-Chloro-3-methyl-r-2,c-6-diphenylpiperidin-4-one Data collection

**Table:**

Rigaku Oxford Diffraction diffractometer	2847 independent reflections
Radiation source: Enhance (Cu) X-ray Source	2456 reflections with *I* > 2σ(*I*)
Graphite monochromator	*R*_int_ = 0.030
Detector resolution: 16.0416 pixels mm^-1^	θ_max_ = 71.6°, θ_min_ = 4.2°
ω scans	*h* = −5→8
Absorption correction: multi-scan CrysAlisPro (Agilent, 2014)	*k* = −13→12
*T*_min_ = 0.609, *T*_max_ = 1.000	*l* = −13→13
4920 measured reflections	

### (I) 3-Chloro-3-methyl-r-2,c-6-diphenylpiperidin-4-one Refinement

**Table:**

Refinement on *F*^2^	Primary atom site location: structure-invariant direct methods
Least-squares matrix: full	Hydrogen site location: mixed
*R*[*F*^2^ > 2σ(*F*^2^)] = 0.057	H atoms treated by a mixture of independent and constrained refinement
*wR*(*F*^2^) = 0.168	*w* = 1/[σ^2^(*F*_o_^2^) + (0.1016*P*)^2^ + 0.3319*P*] where *P* = (*F*_o_^2^ + 2*F*_c_^2^)/3
*S* = 1.05	(Δ/σ)_max_ < 0.001
2847 reflections	Δρ_max_ = 0.68 e Å^−^^3^
195 parameters	Δρ_min_ = −0.29 e Å^−^^3^
0 restraints	

### (I) 3-Chloro-3-methyl-r-2,c-6-diphenylpiperidin-4-one Special details

**Table:**

Geometry. All esds (except the esd in the dihedral angle between two l.s. planes) are estimated using the full covariance matrix. The cell esds are taken into account individually in the estimation of esds in distances, angles and torsion angles; correlations between esds in cell parameters are only used when they are defined by crystal symmetry. An approximate (isotropic) treatment of cell esds is used for estimating esds involving l.s. planes.

### (I) 3-Chloro-3-methyl-r-2,c-6-diphenylpiperidin-4-one Fractional atomic coordinates and isotropic or equivalent isotropic displacement parameters (Å^2^)

**Table:**

	*x*	*y*	*z*	*U*_iso_*/*U*_eq_	
Cl1	0.84481 (9)	0.47065 (6)	0.39680 (5)	0.0398 (2)	
O1	1.0539 (3)	0.6618 (2)	0.10828 (18)	0.0419 (5)	
N1	0.4811 (3)	0.6045 (2)	0.2321 (2)	0.0317 (4)	
H1	0.366 (5)	0.597 (3)	0.221 (3)	0.030 (7)*	
C1	0.8931 (4)	0.6412 (3)	0.1735 (2)	0.0329 (5)	
C2	0.8483 (3)	0.5027 (2)	0.2271 (2)	0.0305 (5)	
C3	0.6344 (3)	0.5032 (2)	0.1920 (2)	0.0301 (5)	
H3	0.6451	0.5259	0.0976	0.036*	
C4	0.5174 (4)	0.7368 (2)	0.1683 (2)	0.0330 (5)	
H4	0.5264	0.7524	0.0747	0.040*	
C5	0.7243 (4)	0.7487 (2)	0.2022 (2)	0.0357 (5)	
H5A	0.7597	0.8344	0.1527	0.043*	
H5B	0.7110	0.7434	0.2932	0.043*	
C6	0.5636 (3)	0.3733 (2)	0.2443 (2)	0.0307 (5)	
C7	0.5880 (4)	0.2906 (3)	0.1680 (2)	0.0355 (5)	
H7	0.6524	0.3150	0.0834	0.043*	
C8	0.5196 (4)	0.1733 (3)	0.2141 (3)	0.0432 (6)	
H8	0.5352	0.1183	0.1605	0.052*	
C9	0.4285 (4)	0.1354 (3)	0.3377 (3)	0.0448 (6)	
H9	0.3834	0.0541	0.3696	0.054*	
C10	0.4040 (4)	0.2172 (3)	0.4142 (3)	0.0441 (6)	
H10	0.3422	0.1915	0.4993	0.053*	
C11	0.4687 (4)	0.3364 (3)	0.3680 (2)	0.0371 (5)	
H11	0.4483	0.3928	0.4207	0.044*	
C12	0.3394 (4)	0.8335 (2)	0.2104 (2)	0.0335 (5)	
C13	0.2480 (4)	0.9433 (3)	0.1241 (3)	0.0422 (6)	
H13	0.2997	0.9597	0.0368	0.051*	
C14	0.0817 (5)	1.0298 (3)	0.1631 (3)	0.0479 (7)	
H14	0.0217	1.1054	0.1031	0.057*	
C15	0.0041 (4)	1.0049 (3)	0.2902 (3)	0.0463 (7)	
H15	−0.1102	1.0630	0.3176	0.056*	
C16	0.0935 (4)	0.8953 (3)	0.3769 (3)	0.0446 (6)	
H16	0.0405	0.8783	0.4640	0.053*	
C17	0.2606 (4)	0.8099 (3)	0.3375 (2)	0.0379 (6)	
H17	0.3214	0.7349	0.3977	0.045*	
C18	1.0159 (4)	0.4010 (3)	0.1837 (3)	0.0369 (5)	
H18A	1.1507	0.4128	0.1957	0.055*	
H18B	1.0120	0.4111	0.0938	0.055*	
H18C	0.9931	0.3136	0.2335	0.055*	

### (I) 3-Chloro-3-methyl-r-2,c-6-diphenylpiperidin-4-one Atomic displacement parameters (Å^2^)

**Table:**

	*U*^11^	*U*^22^	*U*^33^	*U*^12^	*U*^13^	*U*^23^
Cl1	0.0387 (4)	0.0523 (4)	0.0297 (3)	−0.0089 (3)	−0.0114 (2)	−0.0087 (3)
O1	0.0284 (9)	0.0543 (11)	0.0436 (10)	−0.0149 (8)	0.0023 (7)	−0.0132 (8)
N1	0.0201 (9)	0.0363 (11)	0.0398 (11)	−0.0026 (8)	−0.0084 (8)	−0.0110 (8)
C1	0.0272 (11)	0.0446 (13)	0.0297 (11)	−0.0073 (10)	−0.0107 (9)	−0.0098 (10)
C2	0.0235 (11)	0.0418 (13)	0.0261 (10)	−0.0037 (9)	−0.0071 (8)	−0.0087 (9)
C3	0.0233 (10)	0.0382 (12)	0.0292 (11)	−0.0037 (9)	−0.0087 (8)	−0.0083 (9)
C4	0.0286 (11)	0.0374 (12)	0.0330 (12)	−0.0035 (9)	−0.0077 (9)	−0.0094 (9)
C5	0.0309 (12)	0.0384 (13)	0.0397 (13)	−0.0097 (10)	−0.0037 (10)	−0.0114 (10)
C6	0.0206 (10)	0.0384 (12)	0.0347 (12)	−0.0022 (9)	−0.0090 (9)	−0.0115 (10)
C7	0.0250 (11)	0.0434 (13)	0.0395 (13)	−0.0024 (9)	−0.0065 (9)	−0.0146 (11)
C8	0.0356 (13)	0.0429 (14)	0.0568 (16)	−0.0010 (11)	−0.0131 (12)	−0.0223 (12)
C9	0.0362 (13)	0.0405 (14)	0.0582 (17)	−0.0109 (11)	−0.0142 (12)	−0.0069 (12)
C10	0.0397 (14)	0.0519 (16)	0.0413 (14)	−0.0172 (12)	−0.0058 (11)	−0.0069 (12)
C11	0.0324 (12)	0.0452 (14)	0.0358 (12)	−0.0106 (10)	−0.0039 (10)	−0.0120 (10)
C12	0.0273 (11)	0.0379 (12)	0.0377 (12)	−0.0069 (10)	−0.0071 (9)	−0.0113 (10)
C13	0.0419 (14)	0.0427 (14)	0.0406 (14)	−0.0041 (11)	−0.0101 (11)	−0.0093 (11)
C14	0.0411 (14)	0.0416 (14)	0.0591 (17)	0.0059 (11)	−0.0187 (13)	−0.0139 (13)
C15	0.0321 (13)	0.0481 (15)	0.0619 (18)	0.0014 (11)	−0.0083 (12)	−0.0246 (13)
C16	0.0360 (13)	0.0525 (16)	0.0468 (15)	−0.0072 (12)	−0.0004 (11)	−0.0192 (13)
C17	0.0328 (12)	0.0407 (13)	0.0381 (13)	−0.0037 (10)	−0.0055 (10)	−0.0095 (10)
C18	0.0265 (11)	0.0431 (13)	0.0427 (13)	−0.0021 (10)	−0.0075 (10)	−0.0151 (11)

### (I) 3-Chloro-3-methyl-r-2,c-6-diphenylpiperidin-4-one Geometric parameters (Å, º)

**Table:**

Cl1—C2	1.816 (2)	C8—C9	1.384 (4)
O1—C1	1.215 (3)	C9—H9	0.9500
N1—H1	0.83 (3)	C9—C10	1.383 (4)
N1—C3	1.454 (3)	C10—H10	0.9500
N1—C4	1.461 (3)	C10—C11	1.388 (4)
C1—C2	1.529 (4)	C11—H11	0.9500
C1—C5	1.507 (3)	C12—C13	1.385 (4)
C2—C3	1.553 (3)	C12—C17	1.389 (4)
C2—C18	1.520 (3)	C13—H13	0.9500
C3—H3	1.0000	C13—C14	1.391 (4)
C3—C6	1.515 (3)	C14—H14	0.9500
C4—H4	1.0000	C14—C15	1.388 (4)
C4—C5	1.546 (3)	C15—H15	0.9500
C4—C12	1.517 (3)	C15—C16	1.381 (4)
C5—H5A	0.9900	C16—H16	0.9500
C5—H5B	0.9900	C16—C17	1.389 (4)
C6—C7	1.389 (3)	C17—H17	0.9500
C6—C11	1.393 (3)	C18—H18A	0.9800
C7—H7	0.9500	C18—H18B	0.9800
C7—C8	1.381 (4)	C18—H18C	0.9800
C8—H8	0.9500		
			
C3—N1—H1	109 (2)	C7—C8—H8	119.8
C3—N1—C4	114.13 (19)	C7—C8—C9	120.5 (3)
C4—N1—H1	110 (2)	C9—C8—H8	119.8
O1—C1—C2	121.0 (2)	C8—C9—H9	120.4
O1—C1—C5	122.8 (2)	C10—C9—C8	119.3 (3)
C5—C1—C2	116.2 (2)	C10—C9—H9	120.4
C1—C2—Cl1	103.69 (15)	C9—C10—H10	119.7
C1—C2—C3	108.02 (19)	C9—C10—C11	120.7 (3)
C3—C2—Cl1	111.52 (16)	C11—C10—H10	119.7
C18—C2—Cl1	107.95 (16)	C6—C11—H11	120.0
C18—C2—C1	113.27 (19)	C10—C11—C6	119.9 (2)
C18—C2—C3	112.11 (19)	C10—C11—H11	120.0
N1—C3—C2	109.95 (19)	C13—C12—C4	121.4 (2)
N1—C3—H3	107.4	C13—C12—C17	118.8 (2)
N1—C3—C6	110.15 (18)	C17—C12—C4	119.7 (2)
C2—C3—H3	107.4	C12—C13—H13	119.5
C6—C3—C2	114.24 (19)	C12—C13—C14	121.0 (3)
C6—C3—H3	107.4	C14—C13—H13	119.5
N1—C4—H4	109.3	C13—C14—H14	120.2
N1—C4—C5	108.10 (19)	C15—C14—C13	119.5 (3)
N1—C4—C12	109.07 (19)	C15—C14—H14	120.2
C5—C4—H4	109.3	C14—C15—H15	120.1
C12—C4—H4	109.3	C16—C15—C14	119.9 (3)
C12—C4—C5	111.7 (2)	C16—C15—H15	120.1
C1—C5—C4	110.3 (2)	C15—C16—H16	119.8
C1—C5—H5A	109.6	C15—C16—C17	120.3 (3)
C1—C5—H5B	109.6	C17—C16—H16	119.8
C4—C5—H5A	109.6	C12—C17—H17	119.8
C4—C5—H5B	109.6	C16—C17—C12	120.4 (3)
H5A—C5—H5B	108.1	C16—C17—H17	119.8
C7—C6—C3	120.2 (2)	C2—C18—H18A	109.5
C7—C6—C11	119.1 (2)	C2—C18—H18B	109.5
C11—C6—C3	120.7 (2)	C2—C18—H18C	109.5
C6—C7—H7	119.7	H18A—C18—H18B	109.5
C8—C7—C6	120.5 (2)	H18A—C18—H18C	109.5
C8—C7—H7	119.7	H18B—C18—H18C	109.5
			
Cl1—C2—C3—N1	60.5 (2)	C4—C12—C13—C14	178.5 (2)
Cl1—C2—C3—C6	−63.9 (2)	C4—C12—C17—C16	−177.9 (2)
O1—C1—C2—Cl1	113.3 (2)	C5—C1—C2—Cl1	−69.0 (2)
O1—C1—C2—C3	−128.2 (2)	C5—C1—C2—C3	49.4 (3)
O1—C1—C2—C18	−3.4 (3)	C5—C1—C2—C18	174.2 (2)
O1—C1—C5—C4	127.1 (2)	C5—C4—C12—C13	105.9 (3)
N1—C3—C6—C7	137.0 (2)	C5—C4—C12—C17	−76.4 (3)
N1—C3—C6—C11	−41.4 (3)	C6—C7—C8—C9	−1.0 (4)
N1—C4—C5—C1	53.2 (3)	C7—C6—C11—C10	1.3 (4)
N1—C4—C12—C13	−134.6 (2)	C7—C8—C9—C10	0.9 (4)
N1—C4—C12—C17	43.1 (3)	C8—C9—C10—C11	0.3 (4)
C1—C2—C3—N1	−52.8 (2)	C9—C10—C11—C6	−1.4 (4)
C1—C2—C3—C6	−177.23 (19)	C11—C6—C7—C8	−0.1 (3)
C2—C1—C5—C4	−50.6 (3)	C12—C4—C5—C1	173.2 (2)
C2—C3—C6—C7	−98.7 (2)	C12—C13—C14—C15	−0.9 (4)
C2—C3—C6—C11	82.9 (3)	C13—C12—C17—C16	−0.2 (4)
C3—N1—C4—C5	−62.7 (2)	C13—C14—C15—C16	0.6 (4)
C3—N1—C4—C12	175.60 (18)	C14—C15—C16—C17	−0.1 (4)
C3—C6—C7—C8	−178.5 (2)	C15—C16—C17—C12	−0.1 (4)
C3—C6—C11—C10	179.7 (2)	C17—C12—C13—C14	0.7 (4)
C4—N1—C3—C2	63.7 (2)	C18—C2—C3—N1	−178.29 (19)
C4—N1—C3—C6	−169.51 (18)	C18—C2—C3—C6	57.3 (3)

### (I) 3-Chloro-3-methyl-r-2,c-6-diphenylpiperidin-4-one Hydrogen-bond geometry (Å, º)

*Cg*2 and *Cg*3 are the centroids of the C6–C11 and C12–C17 rings, respectively.

**Table:**

*D*—H···*A*	*D*—H	H···*A*	*D*···*A*	*D*—H···*A*
N1—H1···O1^i^	0.83 (3)	2.49 (3)	3.257 (3)	154 (3)
C9—H9···*Cg*3^ii^	0.95	2.97	3.662 (3)	131
C15—H15···*Cg*2^iii^	0.96	2.98	3.861 (3)	155
C18—H18*A*···*Cg*2^iv^	0.98	2.73	3.497 (3)	136

Symmetry codes: (i) *x*−1, *y*, *z*; (ii) *x*, *y*−1, *z*; (iii) *x*−1, *y*+1, *z*; (iv) *x*+1, *y*, *z*.

### (II) 3-Chloro-3-methyl-r-2,c-6-di-p-tolylpiperidin-4-one Crystal data

**Table:**

C_20_H_22_ClNO	*D*_x_ = 1.240 Mg m^−^^3^
*M**_r_* = 327.83	Cu *K*α radiation, λ = 1.54184 Å
Orthorhombic, *P**n**a*2_1_	Cell parameters from 5659 reflections
*a* = 13.0578 (2) Å	θ = 3.9–71.5°
*b* = 22.6513 (4) Å	µ = 1.94 mm^−^^1^
*c* = 5.93756 (8) Å	*T* = 173 K
*V* = 1756.19 (5) Å^3^	, colourless
*Z* = 4	0.32 × 0.18 × 0.08 mm
*F*(000) = 696	

### (II) 3-Chloro-3-methyl-r-2,c-6-di-p-tolylpiperidin-4-one Data collection

**Table:**

Agilent Xcalibur, Eos, Gemini diffractometer	2966 independent reflections
Radiation source: Enhance (Cu) X-ray Source	2873 reflections with *I* > 2σ(*I*)
Graphite monochromator	*R*_int_ = 0.050
Detector resolution: 16.0416 pixels mm^-1^	θ_max_ = 71.4°, θ_min_ = 3.9°
ω scans	*h* = −15→15
Absorption correction: multi-scan CrysAlisPro (Agilent, 2014)	*k* = −24→27
*T*_min_ = 0.724, *T*_max_ = 1.000	*l* = −6→7
11595 measured reflections	

### (II) 3-Chloro-3-methyl-r-2,c-6-di-p-tolylpiperidin-4-one Refinement

**Table:**

Refinement on *F*^2^	Hydrogen site location: mixed
Least-squares matrix: full	H atoms treated by a mixture of independent and constrained refinement
*R*[*F*^2^ > 2σ(*F*^2^)] = 0.034	*w* = 1/[σ^2^(*F*_o_^2^) + (0.0522*P*)^2^ + 0.1354*P*] where *P* = (*F*_o_^2^ + 2*F*_c_^2^)/3
*wR*(*F*^2^) = 0.087	(Δ/σ)_max_ = 0.001
*S* = 1.07	Δρ_max_ = 0.24 e Å^−^^3^
2966 reflections	Δρ_min_ = −0.21 e Å^−^^3^
214 parameters	Absolute structure: Flack *x* determined using 1017 quotients [(*I*^+^)-(*I*^-^)]/[(*I*^+^)+(*I*^-^)] (Parsons *et al.*, 2013)
1 restraint	Absolute structure parameter: −0.010 (13)

### (II) 3-Chloro-3-methyl-r-2,c-6-di-p-tolylpiperidin-4-one Special details

**Table:**

Geometry. All esds (except the esd in the dihedral angle between two l.s. planes) are estimated using the full covariance matrix. The cell esds are taken into account individually in the estimation of esds in distances, angles and torsion angles; correlations between esds in cell parameters are only used when they are defined by crystal symmetry. An approximate (isotropic) treatment of cell esds is used for estimating esds involving l.s. planes.

### (II) 3-Chloro-3-methyl-r-2,c-6-di-p-tolylpiperidin-4-one Fractional atomic coordinates and isotropic or equivalent isotropic displacement parameters (Å^2^)

**Table:**

	*x*	*y*	*z*	*U*_iso_*/*U*_eq_	
Cl1	0.54670 (4)	0.77906 (3)	0.75415 (12)	0.04218 (17)	
O1	0.43096 (11)	0.75024 (7)	0.2382 (4)	0.0378 (4)	
N1	0.71897 (14)	0.72611 (7)	0.4371 (4)	0.0287 (4)	
H1	0.784 (3)	0.7255 (12)	0.419 (6)	0.034*	
C1	0.50913 (16)	0.74057 (10)	0.3414 (4)	0.0310 (4)	
C2	0.56797 (16)	0.79142 (10)	0.4558 (4)	0.0300 (4)	
C3	0.68295 (15)	0.78608 (8)	0.3965 (4)	0.0269 (4)	
H3	0.6902	0.7941	0.2317	0.032*	
C4	0.66920 (16)	0.68266 (9)	0.2913 (4)	0.0303 (5)	
H4	0.6745	0.6960	0.1312	0.036*	
C5	0.55566 (16)	0.68040 (10)	0.3595 (5)	0.0384 (6)	
H5A	0.5185	0.6526	0.2600	0.046*	
H5B	0.5496	0.6659	0.5163	0.046*	
C6	0.75100 (15)	0.82967 (9)	0.5206 (4)	0.0276 (4)	
C7	0.77498 (16)	0.88426 (9)	0.4254 (4)	0.0313 (4)	
H7	0.7474	0.8945	0.2826	0.038*	
C8	0.83845 (17)	0.92371 (9)	0.5363 (4)	0.0337 (5)	
H8	0.8530	0.9609	0.4694	0.040*	
C9	0.88100 (14)	0.90983 (9)	0.7432 (5)	0.0338 (5)	
C10	0.85823 (18)	0.85499 (10)	0.8368 (4)	0.0336 (5)	
H10	0.8873	0.8444	0.9778	0.040*	
C11	0.79398 (15)	0.81549 (9)	0.7278 (4)	0.0306 (4)	
H11	0.7792	0.7784	0.7953	0.037*	
C12	0.72266 (16)	0.62377 (9)	0.3163 (4)	0.0309 (5)	
C13	0.77964 (18)	0.60043 (11)	0.1403 (5)	0.0370 (5)	
H13	0.7821	0.6205	−0.0001	0.044*	
C14	0.83321 (19)	0.54782 (11)	0.1675 (5)	0.0403 (6)	
H14	0.8717	0.5325	0.0449	0.048*	
C15	0.83168 (18)	0.51738 (10)	0.3687 (5)	0.0374 (5)	
C16	0.7745 (2)	0.54068 (11)	0.5437 (5)	0.0424 (6)	
H16	0.7719	0.5203	0.6835	0.051*	
C17	0.7208 (2)	0.59322 (10)	0.5193 (5)	0.0388 (5)	
H17	0.6825	0.6084	0.6424	0.047*	
C18	0.52313 (18)	0.85084 (10)	0.3942 (5)	0.0409 (6)	
H18A	0.4509	0.8521	0.4390	0.061*	
H18B	0.5285	0.8568	0.2311	0.061*	
H18C	0.5609	0.8821	0.4725	0.061*	
C19	0.9515 (2)	0.95208 (12)	0.8652 (6)	0.0476 (7)	
H19A	1.0201	0.9500	0.7986	0.071*	
H19B	0.9552	0.9412	1.0248	0.071*	
H19C	0.9250	0.9924	0.8513	0.071*	
C20	0.8889 (2)	0.46004 (11)	0.3978 (6)	0.0486 (7)	
H20A	0.8438	0.4270	0.3588	0.073*	
H20B	0.9111	0.4561	0.5547	0.073*	
H20C	0.9489	0.4597	0.2987	0.073*	

### (II) 3-Chloro-3-methyl-r-2,c-6-di-p-tolylpiperidin-4-one Atomic displacement parameters (Å^2^)

**Table:**

	*U*^11^	*U*^22^	*U*^33^	*U*^12^	*U*^13^	*U*^23^
Cl1	0.0344 (3)	0.0598 (3)	0.0323 (3)	−0.0046 (2)	0.0068 (3)	−0.0043 (3)
O1	0.0232 (7)	0.0479 (8)	0.0423 (10)	0.0006 (6)	−0.0037 (8)	−0.0050 (9)
N1	0.0199 (8)	0.0283 (9)	0.0380 (11)	−0.0017 (6)	0.0002 (8)	−0.0030 (7)
C1	0.0205 (9)	0.0392 (11)	0.0334 (11)	−0.0025 (8)	0.0030 (8)	−0.0007 (9)
C2	0.0254 (9)	0.0353 (10)	0.0294 (11)	0.0014 (8)	0.0011 (9)	−0.0015 (9)
C3	0.0233 (9)	0.0293 (9)	0.0281 (12)	−0.0024 (7)	0.0014 (8)	−0.0005 (8)
C4	0.0291 (9)	0.0285 (9)	0.0333 (13)	−0.0022 (8)	−0.0022 (8)	−0.0017 (8)
C5	0.0271 (11)	0.0334 (11)	0.0548 (16)	−0.0071 (8)	−0.0067 (10)	−0.0025 (11)
C6	0.0222 (9)	0.0275 (9)	0.0332 (11)	−0.0001 (7)	0.0012 (8)	−0.0019 (9)
C7	0.0294 (10)	0.0313 (10)	0.0333 (12)	0.0007 (8)	0.0011 (9)	0.0033 (9)
C8	0.0308 (10)	0.0264 (9)	0.0439 (14)	−0.0020 (8)	0.0056 (9)	0.0001 (9)
C9	0.0252 (9)	0.0317 (9)	0.0445 (13)	−0.0017 (7)	0.0030 (11)	−0.0086 (10)
C10	0.0308 (10)	0.0352 (10)	0.0347 (11)	0.0008 (8)	−0.0046 (9)	−0.0016 (9)
C11	0.0294 (9)	0.0275 (9)	0.0350 (12)	−0.0011 (7)	−0.0007 (9)	0.0012 (9)
C12	0.0291 (10)	0.0288 (9)	0.0347 (13)	−0.0037 (8)	−0.0046 (8)	−0.0040 (8)
C13	0.0375 (12)	0.0398 (12)	0.0336 (13)	0.0007 (9)	−0.0012 (10)	−0.0021 (10)
C14	0.0371 (12)	0.0412 (12)	0.0427 (15)	0.0034 (10)	0.0001 (10)	−0.0104 (10)
C15	0.0328 (10)	0.0311 (10)	0.0483 (15)	−0.0024 (9)	−0.0098 (10)	−0.0062 (10)
C16	0.0504 (14)	0.0366 (12)	0.0401 (14)	0.0000 (10)	−0.0024 (11)	0.0040 (10)
C17	0.0433 (12)	0.0368 (11)	0.0363 (13)	0.0025 (9)	0.0039 (10)	−0.0025 (10)
C18	0.0307 (10)	0.0357 (11)	0.0561 (17)	0.0036 (9)	−0.0063 (11)	−0.0037 (11)
C19	0.0439 (13)	0.0416 (13)	0.0573 (18)	−0.0119 (10)	−0.0041 (12)	−0.0113 (13)
C20	0.0452 (13)	0.0371 (12)	0.0634 (19)	0.0069 (10)	−0.0101 (13)	−0.0063 (12)

### (II) 3-Chloro-3-methyl-r-2,c-6-di-p-tolylpiperidin-4-one Geometric parameters (Å, º)

**Table:**

Cl1—C2	1.815 (3)	C10—H10	0.9500
O1—C1	1.211 (3)	C10—C11	1.387 (3)
N1—H1	0.85 (3)	C11—H11	0.9500
N1—C3	1.458 (3)	C12—C13	1.388 (3)
N1—C4	1.463 (3)	C12—C17	1.390 (4)
C1—C2	1.542 (3)	C13—H13	0.9500
C1—C5	1.496 (3)	C13—C14	1.391 (3)
C2—C3	1.547 (3)	C14—H14	0.9500
C2—C18	1.513 (3)	C14—C15	1.379 (4)
C3—H3	1.0000	C15—C16	1.385 (4)
C3—C6	1.519 (3)	C15—C20	1.508 (3)
C4—H4	1.0000	C16—H16	0.9500
C4—C5	1.538 (3)	C16—C17	1.389 (4)
C4—C12	1.513 (3)	C17—H17	0.9500
C5—H5A	0.9900	C18—H18A	0.9800
C5—H5B	0.9900	C18—H18B	0.9800
C6—C7	1.395 (3)	C18—H18C	0.9800
C6—C11	1.390 (3)	C19—H19A	0.9800
C7—H7	0.9500	C19—H19B	0.9800
C7—C8	1.385 (3)	C19—H19C	0.9800
C8—H8	0.9500	C20—H20A	0.9800
C8—C9	1.384 (4)	C20—H20B	0.9800
C9—C10	1.393 (3)	C20—H20C	0.9800
C9—C19	1.513 (3)		
			
C3—N1—H1	108.3 (18)	C9—C10—H10	119.4
C3—N1—C4	112.70 (18)	C11—C10—C9	121.2 (2)
C4—N1—H1	111 (2)	C11—C10—H10	119.4
O1—C1—C2	120.5 (2)	C6—C11—H11	119.7
O1—C1—C5	122.9 (2)	C10—C11—C6	120.5 (2)
C5—C1—C2	116.51 (19)	C10—C11—H11	119.7
C1—C2—Cl1	103.80 (15)	C13—C12—C4	120.6 (2)
C1—C2—C3	108.96 (17)	C13—C12—C17	118.2 (2)
C3—C2—Cl1	111.02 (16)	C17—C12—C4	121.1 (2)
C18—C2—Cl1	108.31 (18)	C12—C13—H13	119.7
C18—C2—C1	111.42 (19)	C12—C13—C14	120.6 (2)
C18—C2—C3	112.96 (19)	C14—C13—H13	119.7
N1—C3—C2	110.39 (17)	C13—C14—H14	119.3
N1—C3—H3	107.5	C15—C14—C13	121.4 (2)
N1—C3—C6	109.69 (17)	C15—C14—H14	119.3
C2—C3—H3	107.5	C14—C15—C16	117.9 (2)
C6—C3—C2	114.00 (17)	C14—C15—C20	121.5 (3)
C6—C3—H3	107.5	C16—C15—C20	120.6 (3)
N1—C4—H4	109.1	C15—C16—H16	119.3
N1—C4—C5	107.14 (18)	C15—C16—C17	121.4 (3)
N1—C4—C12	109.27 (18)	C17—C16—H16	119.3
C5—C4—H4	109.1	C12—C17—H17	119.7
C12—C4—H4	109.1	C16—C17—C12	120.6 (3)
C12—C4—C5	112.94 (18)	C16—C17—H17	119.7
C1—C5—C4	110.02 (18)	C2—C18—H18A	109.5
C1—C5—H5A	109.7	C2—C18—H18B	109.5
C1—C5—H5B	109.7	C2—C18—H18C	109.5
C4—C5—H5A	109.7	H18A—C18—H18B	109.5
C4—C5—H5B	109.7	H18A—C18—H18C	109.5
H5A—C5—H5B	108.2	H18B—C18—H18C	109.5
C7—C6—C3	120.7 (2)	C9—C19—H19A	109.5
C11—C6—C3	121.01 (19)	C9—C19—H19B	109.5
C11—C6—C7	118.2 (2)	C9—C19—H19C	109.5
C6—C7—H7	119.6	H19A—C19—H19B	109.5
C8—C7—C6	120.9 (2)	H19A—C19—H19C	109.5
C8—C7—H7	119.6	H19B—C19—H19C	109.5
C7—C8—H8	119.5	C15—C20—H20A	109.5
C9—C8—C7	121.0 (2)	C15—C20—H20B	109.5
C9—C8—H8	119.5	C15—C20—H20C	109.5
C8—C9—C10	118.1 (2)	H20A—C20—H20B	109.5
C8—C9—C19	121.7 (2)	H20A—C20—H20C	109.5
C10—C9—C19	120.2 (3)	H20B—C20—H20C	109.5
			
Cl1—C2—C3—N1	64.2 (2)	C5—C1—C2—Cl1	−72.9 (2)
Cl1—C2—C3—C6	−59.8 (2)	C5—C1—C2—C3	45.4 (3)
O1—C1—C2—Cl1	109.0 (2)	C5—C1—C2—C18	170.7 (2)
O1—C1—C2—C3	−132.7 (2)	C5—C4—C12—C13	129.5 (2)
O1—C1—C2—C18	−7.4 (3)	C5—C4—C12—C17	−54.1 (3)
O1—C1—C5—C4	128.2 (2)	C6—C7—C8—C9	0.9 (3)
N1—C3—C6—C7	143.2 (2)	C7—C6—C11—C10	0.5 (3)
N1—C3—C6—C11	−34.4 (3)	C7—C8—C9—C10	0.1 (3)
N1—C4—C5—C1	56.9 (3)	C7—C8—C9—C19	179.2 (2)
N1—C4—C12—C13	−111.4 (2)	C8—C9—C10—C11	−0.7 (3)
N1—C4—C12—C17	65.1 (3)	C9—C10—C11—C6	0.4 (3)
C1—C2—C3—N1	−49.5 (3)	C11—C6—C7—C8	−1.1 (3)
C1—C2—C3—C6	−173.48 (19)	C12—C4—C5—C1	177.2 (2)
C2—C1—C5—C4	−49.9 (3)	C12—C13—C14—C15	−0.1 (4)
C2—C3—C6—C7	−92.4 (2)	C13—C12—C17—C16	−0.2 (4)
C2—C3—C6—C11	89.9 (2)	C13—C14—C15—C16	0.3 (4)
C3—N1—C4—C5	−66.6 (2)	C13—C14—C15—C20	179.4 (2)
C3—N1—C4—C12	170.74 (18)	C14—C15—C16—C17	−0.5 (4)
C3—C6—C7—C8	−178.9 (2)	C15—C16—C17—C12	0.4 (4)
C3—C6—C11—C10	178.2 (2)	C17—C12—C13—C14	0.0 (3)
C4—N1—C3—C2	64.0 (2)	C18—C2—C3—N1	−173.9 (2)
C4—N1—C3—C6	−169.52 (18)	C18—C2—C3—C6	62.1 (3)
C4—C12—C13—C14	176.6 (2)	C19—C9—C10—C11	−179.9 (2)
C4—C12—C17—C16	−176.7 (2)	C20—C15—C16—C17	−179.6 (2)

### (II) 3-Chloro-3-methyl-r-2,c-6-di-p-tolylpiperidin-4-one Hydrogen-bond geometry (Å, º)

*Cg*2 and *Cg*3 are the centroids of the C6–C11 and C12–C17 rings, respectively.

**Table:**

*D*—H···*A*	*D*—H	H···*A*	*D*···*A*	*D*—H···*A*
N1—H1···O1^i^	0.85 (3)	2.27 (3)	3.057 (2)	154 (3)
C18—H18*A*···*Cg*3^ii^	0.98	2.92	3.686 (3)	135
C20—H20*A*···*Cg*2^iii^	0.97	2.81	3.724 (3)	156

Symmetry codes: (i) *x*+1/2, −*y*+3/2, *z*; (ii) −*x*−1/2, *y*+3/2, *z*+1/2; (iii) *x*+3/2, −*y*−1/2, *z*−1.

### (III) 3-Chloro-3-methyl-r-2,c-6-bis(4-chlorophenyl)piperidin-4-one Crystal data

**Table:**

C_18_H_16_Cl_3_NO	*D*_x_ = 1.419 Mg m^−^^3^
*M**_r_* = 368.67	Cu *K*α radiation, λ = 1.54184 Å
Orthorhombic, *P**n**a*2_1_	Cell parameters from 5579 reflections
*a* = 13.2430 (4) Å	θ = 3.9–71.2°
*b* = 22.3945 (6) Å	µ = 4.83 mm^−^^1^
*c* = 5.81947 (14) Å	*T* = 173 K
*V* = 1725.88 (8) Å^3^	Prism, colourless
*Z* = 4	0.34 × 0.14 × 0.14 mm
*F*(000) = 760	

### (III) 3-Chloro-3-methyl-r-2,c-6-bis(4-chlorophenyl)piperidin-4-one Data collection

**Table:**

Agilent Xcalibur, Eos, Gemini diffractometer	2602 independent reflections
Radiation source: Enhance (Cu) X-ray Source	2494 reflections with *I* > 2σ(*I*)
Graphite monochromator	*R*_int_ = 0.033
Detector resolution: 16.0416 pixels mm^-1^	θ_max_ = 71.4°, θ_min_ = 3.9°
ω scans	*h* = −16→15
Absorption correction: multi-scan CrysAlisPro (Agilent, 2014)	*k* = −27→27
*T*_min_ = 0.646, *T*_max_ = 1.000	*l* = −7→4
12474 measured reflections	

### (III) 3-Chloro-3-methyl-r-2,c-6-bis(4-chlorophenyl)piperidin-4-one Refinement

**Table:**

Refinement on *F*^2^	Hydrogen site location: mixed
Least-squares matrix: full	H atoms treated by a mixture of independent and constrained refinement
*R*[*F*^2^ > 2σ(*F*^2^)] = 0.032	*w* = 1/[σ^2^(*F*_o_^2^) + (0.0469*P*)^2^ + 0.6568*P*] where *P* = (*F*_o_^2^ + 2*F*_c_^2^)/3
*wR*(*F*^2^) = 0.084	(Δ/σ)_max_ = 0.001
*S* = 1.02	Δρ_max_ = 0.45 e Å^−^^3^
2602 reflections	Δρ_min_ = −0.23 e Å^−^^3^
212 parameters	Absolute structure: Flack *x* determined using 695 quotients [(*I*^+^)-(*I*^-^)]/[(*I*^+^)+(*I*^-^)] (Parsons *et al.*, 2013)
1 restraint	Absolute structure parameter: 0.135 (13)

### (III) 3-Chloro-3-methyl-r-2,c-6-bis(4-chlorophenyl)piperidin-4-one Special details

**Table:**

Geometry. All esds (except the esd in the dihedral angle between two l.s. planes) are estimated using the full covariance matrix. The cell esds are taken into account individually in the estimation of esds in distances, angles and torsion angles; correlations between esds in cell parameters are only used when they are defined by crystal symmetry. An approximate (isotropic) treatment of cell esds is used for estimating esds involving l.s. planes.

### (III) 3-Chloro-3-methyl-r-2,c-6-bis(4-chlorophenyl)piperidin-4-one Fractional atomic coordinates and isotropic or equivalent isotropic displacement parameters (Å^2^)

**Table:**

	*x*	*y*	*z*	*U*_iso_*/*U*_eq_	
Cl1	0.45288 (4)	0.77767 (3)	0.75233 (13)	0.04111 (16)	
Cl2	0.03876 (5)	0.95332 (3)	0.88033 (17)	0.05145 (19)	
Cl3	0.10150 (6)	0.45148 (3)	0.38178 (19)	0.0578 (2)	
O1	0.57167 (12)	0.74818 (9)	0.2363 (4)	0.0363 (4)	
N1	0.28424 (14)	0.72489 (9)	0.4198 (4)	0.0288 (5)	
H1	0.228 (2)	0.7238 (12)	0.413 (6)	0.035*	
C1	0.49247 (17)	0.73891 (11)	0.3332 (5)	0.0294 (6)	
C2	0.43340 (17)	0.79041 (11)	0.4461 (5)	0.0277 (5)	
C3	0.32060 (17)	0.78557 (9)	0.3839 (5)	0.0261 (5)	
H3	0.3143	0.7944	0.2161	0.031*	
C4	0.33396 (17)	0.68095 (10)	0.2728 (5)	0.0291 (5)	
H4	0.3302	0.6946	0.1095	0.035*	
C5	0.44518 (17)	0.67810 (12)	0.3470 (6)	0.0364 (6)	
H5A	0.4497	0.6630	0.5066	0.044*	
H5B	0.4823	0.6501	0.2460	0.044*	
C6	0.25273 (17)	0.82914 (10)	0.5104 (5)	0.0260 (5)	
C7	0.22816 (18)	0.88418 (11)	0.4167 (5)	0.0303 (6)	
H7	0.2565	0.8955	0.2733	0.036*	
C8	0.1631 (2)	0.92294 (11)	0.5281 (5)	0.0335 (6)	
H8	0.1467	0.9606	0.4625	0.040*	
C9	0.12236 (17)	0.90591 (11)	0.7364 (6)	0.0336 (6)	
C10	0.14495 (18)	0.85140 (11)	0.8344 (5)	0.0321 (6)	
H10	0.1161	0.8402	0.9776	0.039*	
C11	0.21035 (17)	0.81329 (11)	0.7205 (5)	0.0303 (6)	
H11	0.2265	0.7757	0.7869	0.036*	
C12	0.27945 (18)	0.62177 (11)	0.2956 (5)	0.0312 (6)	
C13	0.2849 (2)	0.58865 (14)	0.4970 (6)	0.0445 (7)	
H13	0.3268	0.6021	0.6190	0.053*	
C14	0.2303 (3)	0.53636 (14)	0.5229 (7)	0.0471 (8)	
H14	0.2346	0.5140	0.6613	0.057*	
C15	0.1701 (2)	0.51733 (11)	0.3470 (6)	0.0402 (7)	
C16	0.1625 (2)	0.54859 (14)	0.1446 (6)	0.0420 (7)	
H16	0.1206	0.5347	0.0235	0.050*	
C17	0.2180 (2)	0.60143 (13)	0.1208 (6)	0.0384 (7)	
H17	0.2133	0.6236	−0.0179	0.046*	
C18	0.47925 (19)	0.85094 (11)	0.3858 (6)	0.0356 (6)	
H18A	0.4427	0.8825	0.4675	0.053*	
H18B	0.4740	0.8576	0.2198	0.053*	
H18C	0.5505	0.8516	0.4313	0.053*	

### (III) 3-Chloro-3-methyl-r-2,c-6-bis(4-chlorophenyl)piperidin-4-one Atomic displacement parameters (Å^2^)

**Table:**

	*U*^11^	*U*^22^	*U*^33^	*U*^12^	*U*^13^	*U*^23^
Cl1	0.0355 (3)	0.0587 (4)	0.0292 (3)	0.0045 (3)	−0.0049 (3)	−0.0023 (3)
Cl2	0.0479 (3)	0.0469 (3)	0.0596 (5)	0.0179 (3)	0.0108 (4)	−0.0101 (4)
Cl3	0.0598 (4)	0.0405 (3)	0.0732 (5)	−0.0189 (3)	0.0166 (4)	−0.0065 (4)
O1	0.0232 (7)	0.0470 (9)	0.0388 (10)	−0.0017 (7)	0.0030 (9)	−0.0059 (11)
N1	0.0196 (8)	0.0281 (9)	0.0386 (13)	0.0013 (7)	0.0008 (9)	−0.0027 (10)
C1	0.0220 (10)	0.0378 (12)	0.0286 (13)	0.0045 (9)	−0.0038 (10)	−0.0023 (11)
C2	0.0252 (10)	0.0342 (11)	0.0235 (12)	0.0000 (9)	0.0010 (10)	−0.0018 (10)
C3	0.0267 (10)	0.0265 (10)	0.0252 (13)	0.0012 (8)	−0.0026 (10)	0.0008 (11)
C4	0.0305 (10)	0.0251 (10)	0.0318 (14)	0.0010 (9)	0.0013 (11)	−0.0013 (11)
C5	0.0260 (11)	0.0354 (12)	0.0478 (16)	0.0062 (9)	0.0070 (12)	−0.0021 (13)
C6	0.0219 (9)	0.0280 (11)	0.0282 (12)	−0.0016 (9)	−0.0020 (9)	−0.0018 (11)
C7	0.0311 (11)	0.0279 (11)	0.0318 (14)	0.0007 (9)	0.0013 (10)	0.0001 (11)
C8	0.0337 (12)	0.0262 (11)	0.0406 (16)	0.0047 (10)	−0.0027 (12)	0.0011 (12)
C9	0.0270 (10)	0.0320 (12)	0.0418 (15)	0.0038 (9)	−0.0016 (12)	−0.0085 (13)
C10	0.0287 (11)	0.0375 (13)	0.0302 (14)	0.0001 (9)	0.0051 (10)	−0.0018 (12)
C11	0.0286 (10)	0.0282 (11)	0.0340 (15)	0.0019 (9)	0.0021 (11)	0.0020 (11)
C12	0.0309 (11)	0.0270 (11)	0.0357 (16)	0.0027 (9)	0.0047 (10)	−0.0018 (11)
C13	0.0550 (16)	0.0411 (15)	0.0375 (17)	−0.0085 (13)	−0.0069 (14)	0.0021 (14)
C14	0.0615 (18)	0.0365 (14)	0.0434 (18)	−0.0062 (13)	−0.0018 (16)	0.0052 (15)
C15	0.0383 (12)	0.0304 (12)	0.0519 (17)	−0.0028 (10)	0.0133 (13)	−0.0090 (13)
C16	0.0382 (13)	0.0431 (15)	0.0446 (17)	−0.0045 (12)	0.0007 (13)	−0.0089 (13)
C17	0.0387 (13)	0.0385 (14)	0.0379 (16)	−0.0017 (11)	−0.0008 (13)	0.0004 (13)
C18	0.0297 (11)	0.0321 (11)	0.0449 (16)	−0.0036 (9)	0.0045 (12)	−0.0012 (14)

### (III) 3-Chloro-3-methyl-r-2,c-6-bis(4-chlorophenyl)piperidin-4-one Geometric parameters (Å, º)

**Table:**

Cl1—C2	1.823 (3)	C7—C8	1.385 (4)
Cl2—C9	1.748 (3)	C8—H8	0.9500
Cl3—C15	1.744 (3)	C8—C9	1.380 (4)
O1—C1	1.209 (3)	C9—C10	1.380 (4)
N1—H1	0.74 (3)	C10—H10	0.9500
N1—C3	1.457 (3)	C10—C11	1.385 (4)
N1—C4	1.460 (3)	C11—H11	0.9500
C1—C2	1.541 (3)	C12—C13	1.389 (4)
C1—C5	1.501 (4)	C12—C17	1.380 (4)
C2—C3	1.541 (3)	C13—H13	0.9500
C2—C18	1.526 (3)	C13—C14	1.384 (4)
C3—H3	1.0000	C14—H14	0.9500
C3—C6	1.517 (3)	C14—C15	1.365 (5)
C4—H4	1.0000	C15—C16	1.374 (5)
C4—C5	1.536 (3)	C16—H16	0.9500
C4—C12	1.515 (3)	C16—C17	1.400 (4)
C5—H5A	0.9900	C17—H17	0.9500
C5—H5B	0.9900	C18—H18A	0.9800
C6—C7	1.386 (3)	C18—H18B	0.9800
C6—C11	1.391 (4)	C18—H18C	0.9800
C7—H7	0.9500		
			
C3—N1—H1	111 (2)	C7—C8—H8	120.6
C3—N1—C4	113.3 (2)	C9—C8—C7	118.7 (2)
C4—N1—H1	113 (2)	C9—C8—H8	120.6
O1—C1—C2	120.7 (2)	C8—C9—Cl2	120.0 (2)
O1—C1—C5	122.9 (2)	C10—C9—Cl2	118.5 (2)
C5—C1—C2	116.4 (2)	C10—C9—C8	121.5 (2)
C1—C2—Cl1	103.17 (17)	C9—C10—H10	120.6
C1—C2—C3	109.8 (2)	C9—C10—C11	118.9 (3)
C3—C2—Cl1	110.85 (17)	C11—C10—H10	120.6
C18—C2—Cl1	107.91 (19)	C6—C11—H11	119.5
C18—C2—C1	111.4 (2)	C10—C11—C6	121.0 (2)
C18—C2—C3	113.2 (2)	C10—C11—H11	119.5
N1—C3—C2	110.62 (19)	C13—C12—C4	121.1 (2)
N1—C3—H3	107.3	C17—C12—C4	120.3 (2)
N1—C3—C6	109.5 (2)	C17—C12—C13	118.5 (2)
C2—C3—H3	107.3	C12—C13—H13	119.4
C6—C3—C2	114.5 (2)	C14—C13—C12	121.1 (3)
C6—C3—H3	107.3	C14—C13—H13	119.4
N1—C4—H4	109.1	C13—C14—H14	120.4
N1—C4—C5	107.2 (2)	C15—C14—C13	119.1 (3)
N1—C4—C12	108.9 (2)	C15—C14—H14	120.4
C5—C4—H4	109.1	C14—C15—Cl3	118.7 (3)
C12—C4—H4	109.1	C14—C15—C16	121.8 (3)
C12—C4—C5	113.3 (2)	C16—C15—Cl3	119.5 (2)
C1—C5—C4	110.3 (2)	C15—C16—H16	120.8
C1—C5—H5A	109.6	C15—C16—C17	118.5 (3)
C1—C5—H5B	109.6	C17—C16—H16	120.8
C4—C5—H5A	109.6	C12—C17—C16	121.0 (3)
C4—C5—H5B	109.6	C12—C17—H17	119.5
H5A—C5—H5B	108.1	C16—C17—H17	119.5
C7—C6—C3	121.3 (2)	C2—C18—H18A	109.5
C7—C6—C11	118.5 (2)	C2—C18—H18B	109.5
C11—C6—C3	120.1 (2)	C2—C18—H18C	109.5
C6—C7—H7	119.4	H18A—C18—H18B	109.5
C8—C7—C6	121.3 (3)	H18A—C18—H18C	109.5
C8—C7—H7	119.4	H18B—C18—H18C	109.5
			
Cl1—C2—C3—N1	−65.4 (2)	C4—C12—C13—C14	176.1 (3)
Cl1—C2—C3—C6	59.0 (2)	C4—C12—C17—C16	−176.2 (2)
Cl2—C9—C10—C11	179.4 (2)	C5—C1—C2—Cl1	74.3 (2)
Cl3—C15—C16—C17	179.8 (2)	C5—C1—C2—C3	−44.0 (3)
O1—C1—C2—Cl1	−106.9 (3)	C5—C1—C2—C18	−170.2 (2)
O1—C1—C2—C3	134.8 (3)	C5—C4—C12—C13	49.2 (4)
O1—C1—C2—C18	8.6 (4)	C5—C4—C12—C17	−134.8 (3)
O1—C1—C5—C4	−129.9 (3)	C6—C7—C8—C9	0.0 (4)
N1—C3—C6—C7	−142.0 (2)	C7—C6—C11—C10	0.1 (4)
N1—C3—C6—C11	35.4 (3)	C7—C8—C9—Cl2	−179.3 (2)
N1—C4—C5—C1	−56.5 (3)	C7—C8—C9—C10	−0.2 (4)
N1—C4—C12—C13	−69.9 (3)	C8—C9—C10—C11	0.3 (4)
N1—C4—C12—C17	106.0 (3)	C9—C10—C11—C6	−0.2 (4)
C1—C2—C3—N1	48.0 (3)	C11—C6—C7—C8	0.1 (4)
C1—C2—C3—C6	172.4 (2)	C12—C4—C5—C1	−176.6 (2)
C2—C1—C5—C4	48.9 (3)	C12—C13—C14—C15	−0.1 (5)
C2—C3—C6—C7	93.1 (3)	C13—C12—C17—C16	−0.2 (4)
C2—C3—C6—C11	−89.6 (3)	C13—C14—C15—Cl3	−179.9 (2)
C3—N1—C4—C5	66.1 (3)	C13—C14—C15—C16	0.2 (5)
C3—N1—C4—C12	−170.9 (2)	C14—C15—C16—C17	−0.3 (4)
C3—C6—C7—C8	177.4 (2)	C15—C16—C17—C12	0.3 (4)
C3—C6—C11—C10	−177.4 (2)	C17—C12—C13—C14	0.1 (4)
C4—N1—C3—C2	−62.9 (3)	C18—C2—C3—N1	173.2 (2)
C4—N1—C3—C6	169.9 (2)	C18—C2—C3—C6	−62.4 (3)

### (III) 3-Chloro-3-methyl-r-2,c-6-bis(4-chlorophenyl)piperidin-4-one Hydrogen-bond geometry (Å, º)

*Cg*3 is the centroid of the C12–C17 ring.

**Table:**

*D*—H···*A*	*D*—H	H···*A*	*D*···*A*	*D*—H···*A*
N1—H1···O1^i^	0.74 (3)	2.40 (3)	3.071 (3)	151 (3)
C10—H10···O1^ii^	0.95	2.56	3.374 (3)	144
C18—H18*C*···*Cg*3^iii^	0.98	2.98	3.725 (3)	134

Symmetry codes: (i) *x*−1/2, −*y*+3/2, *z*; (ii) *x*−1/2, −*y*+3/2, *z*+1; (iii) −*x*+1/2, *y*+3/2, *z*+1/2.
